# State of the European Union’s early notification obligation for drug shortages: enforcement and compliance in eight European countries (2020–2022)

**DOI:** 10.1186/s40545-023-00646-w

**Published:** 2023-11-07

**Authors:** Reko Ravela, Marja Airaksinen, Alan Lyles

**Affiliations:** 1 Clinical Pharmacy Group, Division of Pharmacology and Pharmacotherapy, Faculty of Pharmacy, University of Helsinki, PL 56, Viikinkaari 5, 00014 Helsinki, Finland; 2 School of Health and Human Services, College of Public Affairs, University of Baltimore, Baltimore, USA

## Abstract

**Background:**

Early notification of impending drug shortages is essential for mitigating or preventing shortages. Since 2019 pharmaceutical companies in the European Union (EU) and European Economic Area (EEA) must notify authorities of drug shortages at least two months in advance. This study’s aim was to investigate how advance notification of pharmaceutical shortages is functioning in EU/EEA countries and factors possibly associated with differences in notification times.

**Methods:**

This was a retrospective register study using data from publicly available drug shortage registers of all national authorities in the EU/EEA area having such a register. Actual notification times for all drug shortages during January 2020–November 2022 were calculated and included in the descriptive quantitative analysis.

**Results:**

Data from eight countries (Belgium, Croatia, Finland, Germany, Norway, Slovakia, Slovenia, and Sweden) were available (18,987 notifications in total). Only 5.2% of all shortage notifications were made at least 60 days in advance and 56.2% of all notifications were made on the shortage’s starting day or even later. Data on production-related shortages were available in Belgium, Croatia, Germany, and Norway (*n* = 2097 showing that 3.9% of those shortages were notified at least 60 days in advance, but 74.3% were made on the starting day or even later. The longest advance notification times for drug shortages were found in Finland during a 12-month period in June 2021–May 2022 when progressive notification fees were in effect. During this national policy experiment, 20.0% of the shortages (*n* = 1754) were notified at least 60 days in advance, while 24.9% of the notifications occurred on the starting day or even later. Data on notification times for permanent market withdrawals of drugs were available in three countries (Belgium, Slovenia, and Slovakia, *n* = 1737): 21.2% of these notifications were made at least 60 days in advance, while 45.5% of the notifications occurred on the starting day or even later.

**Conclusions:**

The EU regulatory requirement adopted in 2019 for early notification of drug shortages was unsuccessful in the eight countries having openly available statistics for follow-up. The national policy experiment in Finland with a progressive notification fee seemed to increase compliance with early notification.

## Background

Drug shortages have increased substantially since the start of the 2010s and have become an unresolved challenge for healthcare systems and patients worldwide [1–3]. Defining a shortage as a “serious threat to the right to essential medical treatment” [1], the European Parliament declared in 2020 that the “longstanding problem of shortages of medicines within the EU has worsened exponentially in recent years.” [4]

Multiple stakeholders have agreed (i) on the need for transparency in the medicines supply chain and, (ii) specifically, early notification of impending shortages as essential measures for the mitigation and prevention of inadequate medicines supply. These strategies and measures have been expressed by scientists [5–9], practising health care providers, such as hospital and community pharmacists [10, 11], as well as in policy papers and reports [1, 3, 12, 13].

The flagship initiatives of the EU's Pharmaceutical Strategy [14] to tackle the challenge of drug shortages includes more substantial obligations for pharmaceutical companies to provide earlier notification of impending medication shortages and of withdrawal of products from the market. However, national authorities have reported that implementation of early notification has yet to meet existing EU goals [1, 15, 16]. The aim of this study was to investigate how the early notification system of pharmaceutical shortages in EU/EEA countries that was adopted in 2019 is functioning and factors possibly associated with differences in notification times.

### Importance of early notification

Early notification of impending shortages may have unintended and adverse effects, such as triggering medication hoarding by hospitals, pharmacies, and patients. These could then exacerbate the shortage(s). However, it is possible to minimise these adverse effects by limiting excessive buying, as has been occasionally done in Finland in recent years, both at the wholesaler and the pharmacy level.

More importantly, however, early notification makes it possible for national authorities, wholesalers, and manufacturers to react by expediting the availability of alternative products. Drug shortages are mostly national, rather than global, disruptions [1, 23]; importing substitutes, or even the same product, is often a viable option. The early notification also gives time for gradual and prepared transition(s) to other treatment options and the possibility to prioritise using existing stocks for patients with no good alternatives or in the most critical need.

## Methods

### The legal framework of an EU early notification system

The EU Medicines Directive 2001/83/EC defines the public service obligation for marketing authorisation holders to “*ensure appropriate and continued supplies of… medicinal product”* (paragraph 81). Nevertheless, specific requirements for this public service obligation remains to be defined operationally in both EU and national legislation. Currently, the most clearly defined consequence of that public service obligation is the requirement that market authorisation holders give notice of an impending suspension of supply (paragraph 23):

*“If the product ceases to be placed on the market of a Member State, either temporarily or permanently, the marketing authorisation holder shall notify the competent authority of that Member State. Such notification shall, other than in exceptional circumstances, be made no less than two months before the interruption in the placing on the market of the product.”* [15]

A marketing authorisation holder’s obligation to notify at least two months in advance was codified in 2019. However, some countries have stricter regulations, mandating notification from 3 to 12 months in advance at least for some drugs [1, 17].

Of the EU/EEA countries investigated in our study, Finland, Sweden, Croatia, Norway, Slovenia, and Slovenia have a legal requirement for market authorisation holders to give 2 months advance notification [1]. In Germany, the minimum advance notification time stipulated by law is 6 months for “foreseeable shortages”, and in Belgium, the decreed notification time for reimbursed medicines is 6 months [1, 22].

There is no specified sanction for failure to comply with this EU directive, and to the extent that sanctions do exist, they are under national rather than EU legislation. Though sanctions in the form of administrative fines may be possible, their use is very limited in practice. For example, the EU report “Future-proofing pharmaceutical legislation” [1] notes France and the Netherlands as examples of countries where pharmaceutical companies were fined for negligence of their obligation regarding the continuous supply of medicines. However, in each country only two companies were fined during a year, and it is not known if those cases were related to belated notification.

### The legal framework in Finland

Starting on June 1, 2021, the Finnish Medicines Agency introduced processing fees for shortage notifications [18]. The shortage notification fee amount was calibrated to the lead time duration to encourage early reporting, thereby increasing the time between receipt of the notification and the start of the shortage. For example, marketing authorisation holder notifications made at least 2 months in advance were charged 70 euros. By contrast, notifications made less than 2 weeks before the start of a shortage were charged 1000 euros.

Progressive fees were, however, opposed by the pharmaceutical industry [17]. In May 2022, the Chancellor of Justice in Finland overruled the imposition of progressive fees, determining that the Finnish Medicines Agency (Fimea) had overstepped its authority in ordering punitive costs without having a sufficient legal basis [20]. After this ruling by the Chancellor of Justice, Fimea ceased charging progressive fees and, starting on September 1, 2022, introduced a flat processing fee of 280 euros for all shortage notifications.

### Selection of registers and delimitation of data

We searched notification data from 25 websites maintained by national medicines authorities in the EU/EAA. The European Medicines Agency (EMA) identified these as primary sources for pharmaceutical shortage data [21]. Seventeen (17) national register sites lacked notification dates (date of first notification and the starting date of a shortage).

Therefore, our study includes data from the remaining eight national register sites that had adequate data: Belgium, Croatia, Finland, Germany, Norway, Slovakia, Slovenia and Sweden. Our study included all notifications from these eight national registers made during the period of January 2020–November 2022 with the exception of Belgium, where data were available from the period of July 2022–November 2022. All register data were retrieved between the 5th and 15th of November 2022.

Not all of the registers in the eight nations included in the study contained veterinary drug notifications. Thus, veterinary medicines were excluded from our study to reduce potential bias. Notifications about market entrances and the ending of shortages were excluded. So too were notifications that represented updates after a first notification of the same shortage. In cases where the registers had multiple notifications of the same shortage, we included only the first notification.

### Distinguishing temporary shortages versus permanent market withdrawals

Our primary focus was notifications of temporary drug shortages. However, some registers (Belgium, Croatia, Norway, Slovenia, and Slovakia) included notifications of both permanent and temporary product discontinuations. From a patient and healthcare perspective, permanent market withdrawals can have similar effects as temporary supply shortages. According to the EU directive, the obligation for early notification is similar for both temporary shortages and permanent withdrawals [1].

However, permanent market withdrawals are usually based on company decisions. Therefore, they should be more predictable and thus differ from temporary drug shortage notifications. Based on this, we separated permanent market withdrawals from data as far as it was possible and included them only in a separate analysis.

As the registers differed by country, we made the following separations of the notifications related to temporary notifications and permanent market withdrawals: in Belgium, Slovakia, and Slovenia, we separated temporary notifications and permanent market withdrawals for separate analysis based on their register data classifications. In Croatia and Norway, an explicit classification for the types of supply discontinuation was missing; consequently, all their cases were analysed as temporary shortage notifications. In Finland, Germany and Sweden, notifications of market withdrawals were not included in the registers, and thus, could not be included in the analysis.

### Separation of production-based shortages

Four countries’ registers (Belgium, Germany, Norway, and Croatia) included the pharmaceutical companies’ reasons for the anticipated shortages. In addition, the German registry classified shortages by the notified cause as production-based and others. We emulated this classification with data from the other three countries to investigate whether shortages caused by production problems had been notified earlier than others.

### Data analysis

We calculated the advance notification time in days for each shortage notification based on the first notification date and the starting shortage date in the register. To better understand the distribution of notification times, we sorted notifications into the following three groups by the time between notification and the start of the shortage: (1) at least 60 days before the start of the shortage; (2) 1–59 days before the start of the shortage; and (3) on the same day as the start of the shortage, or later.

### Time series of monthly median notification times

A time series of monthly median times for advance notifications was calculated for each country. From this analysis, Croatia had to be excluded due to the small number of notifications, and Belgium due to its data only starting from July 2022.

## Results

**Fig. 1:**
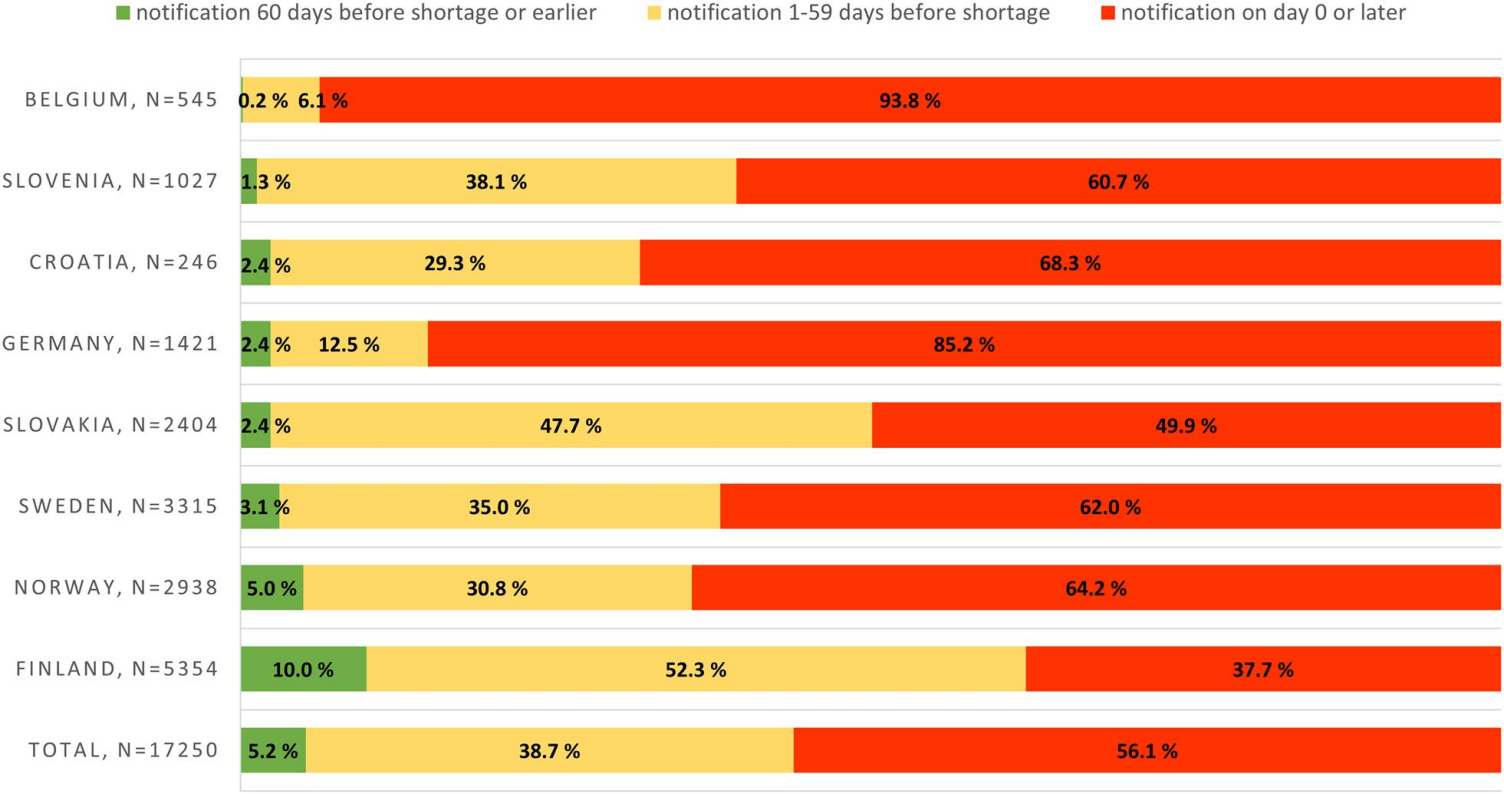
Temporary drug shortage notification times in days by country during January 2020–November 2022, with the exception of Belgium where the observation period was July 2022–November 2022. Permanent market withdrawals not included

In our final dataset, we had 17,250 temporary drug shortage notifications and 1737 notifications for permanent drug product withdrawals. Of the temporary drug shortages, 5.2% were notified at least 60 days in advance as required, and most of the shortages (56.1%) were reported only on the day the shortage began, or even weeks or months later (Fig. 1).

Legally required longer lead time for shortage notifications did not correlate with longer actual notification times. On the contrary, despite the six months requirement, in Germany 85%, and in Belgium 93% of notifications were made on the day the shortage started or later. These were the most extreme data among the countries compared in our research.

### Early notification of temporary shortages reportedly due to a production problem

**Fig. 2:**
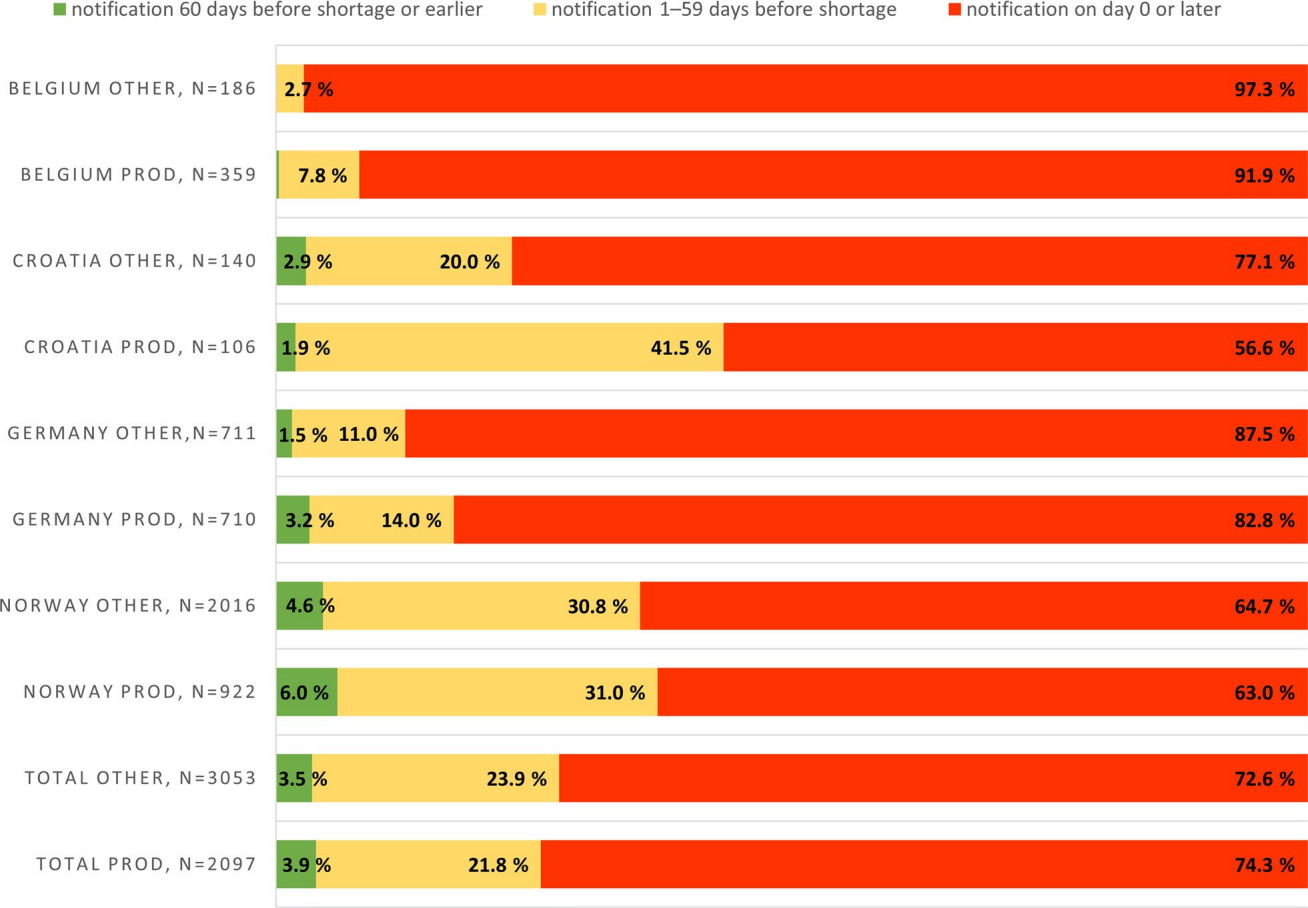
Temporary drug shortage notification times for production-based vs. other temporary shortages (production-related shortage notification data separated in data for Croatia, Germany, and Norway, observation period January 2020–November 2022, for Belgium July 2022–November 2022). PROD = production-related temporary drug shortages; OTHER = temporary drug shortages caused by other than production-related reasons

In the analysis of early notification of shortages caused by production problems, 3.9% were notified at least 60 days in advance as required, and 74.3% of notifications were made on the day the shortage started or later. Of the other, presumably more demand-driven shortages in the same countries, 3.5% were notified at least 60 days in advance as required, and 72.6% of notifications were made on the day the shortage started or later (Fig. 2).

### Case analysis of Finland: improved notification times were associated with progressive notification fees

Among the eight countries studied, Finland had the highest proportion (10.0%) of shortage notifications made at least 60 days in advance and the lowest proportion (37.7%) of notifications made on day the shortage started or later. Finnish data were analysed based on our knowledge of changes in the notification fees during the study period.

**Fig. 3:**
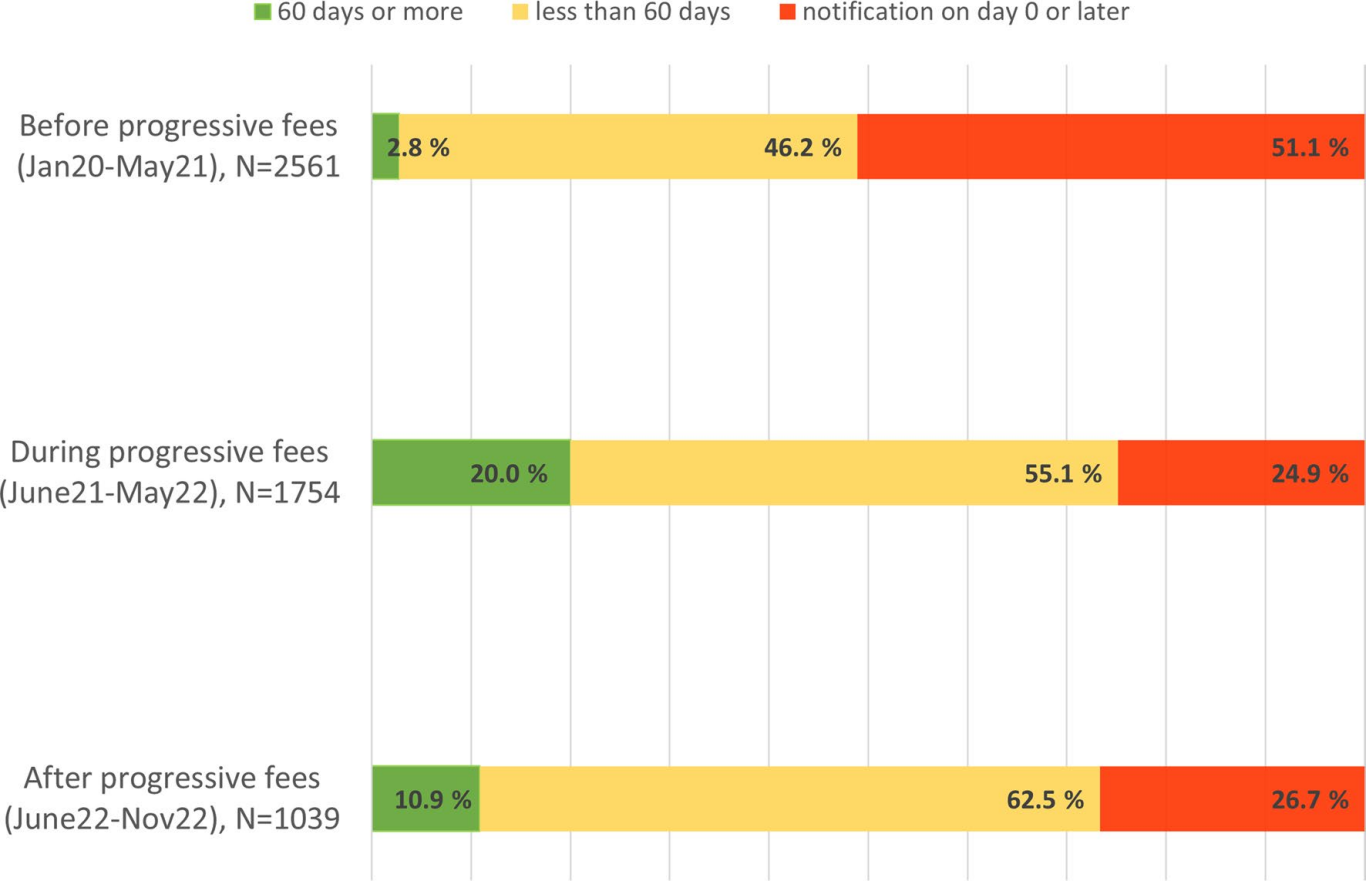
Notification times of temporary drug shortages before, during and after the period of using the progressive notification fees in Finland, observation period in total from January 2020 to November 2022

In the period when progressive notification fees were in use (June 2021–May 2022), the median notification time in Finland improved 17 days in comparison to the prior period (Fig. 3). The share of notifications made at least 60 days in advance rose from 3 to 20%. In months after the end of the policy experiment, notification times were still longer than before the introduction of progressive fees, but the trend was for decreasing notification times.

### Time series of monthly median notification times

**Fig. 4:**
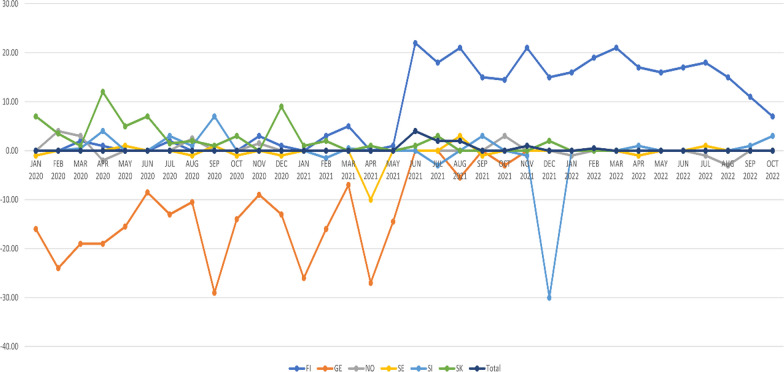
Monthly median notification times in days for temporary drug shortages in Finland, Germany, Norway, Sweden, Slovakia, Slovenia, observation period January 2020–October 2022. In Finland, the progressive notification fees were in effect between June 2021 and May 2022. Permanent market withdrawals not included. (FI-Finland, GE = Germany, NO-Norway, SE = Sweden, SI = Slovenia, SK = Slovenia)

In time series of monthly median shortage notification times, neither general improvement nor deterioration was observed between January 2020 and October 2022 (Fig. 4). However, the following observations were made:

1. A temporary improvement was associated with progressive fees in Finland, clearly diverging from other countries in same time frame.
2. The median notification time of 0 days (meaning notification on the starting day of the shortage) seemed to be level that median notification times were stabilising. Countries having better median notification times at beginning of the time series (Norway and Slovenia) deteriorated to this level, and Germany, having worse median notification times at beginning of the time series, improved to this level.

### Notifications of permanent market withdrawals

**Fig. 5:**
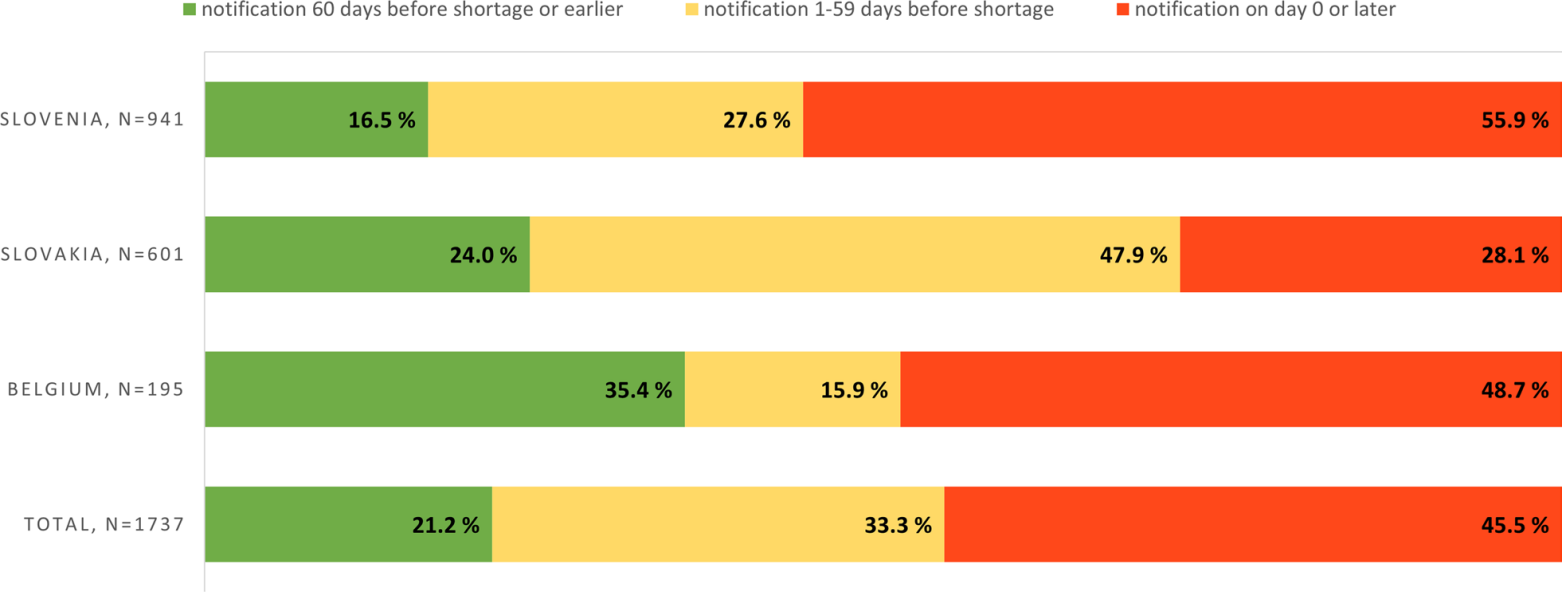
Notification times for permanent market withdrawals (Slovenia, Slovakia January 2020–November 2022), Belgium July 2022–November 2022)

In analysis of permanent market withdrawals (*n* = 1737), 21.2% of notifications fulfilled the obligation of 60 days notification period, though almost half (45.5%) were notified only on the day that distribution was ended or afterward (Fig. 5).

## Discussion

### Compliance with the EU regulatory requirement

Based on our results, implementation of an early notification system for drug shortages is yet to be seen in the EU. Early notification as a routine was not realised in any of the EU/EEA countries that had published data on notification times. The similarity of results across the eight countries studied implies that these results might be generalised to the EU area.

Unlike sudden demand spikes or distribution problems, production disruptions should affect pharmacy supply only after considerable delay. Based on this, the potential and practice for early notification should be better in this group of shortages. However, according to the register data, barely any differences were found in notification with these clearly supply-driven shortages and other shortages that were more demand-driven.

Finland’s policy experiment with progressive fees showed promising results for improving compliance with the early notification requirement. Progressive fees offer a regulatory natural experiment in sanctioning belated notifications. Although the fee levels were moderate, they created a clear and consistent incentive for earlier notification of drug shortages.

In most cases, market withdrawals of drug products are planned decisions based mainly on commercial considerations. Accordingly, lead times from notification to actual market withdrawal were found to be marginally better in this group of notifications, compared with the shortages. However, even these notifications largely failed to meet the requirement of 2 months advance notification.

Currently, notification times appear to be determined more by the concerns of pharmaceutical companies rather than a regulatory requirement or public health needs. From a pharmaceutical company's point of view, most mitigation efforts triggered by an early shortage warning might be seen as threatening the product's commercial prospects as it may alert and trigger movements to a substitute, available product.

Early reporting would also have a disincentive if it led to decreasing demand and increasing competition for the manufacturer’s product post-shortage market. Without the enforcement of regulatory pressure or consequences, it is unrealistic to assume that companies would act against their interests and adopt early notification as a routine practice.

### Policy implications

There has not been an open discussion of the widespread deviation of actual notification times from the early notification requirement that our results show to be a common real-life practice within EU/EEA countries. Actions to remedy national and EU legislative consequences are needed to correct the problem. The legal requirement for companies at least to share information about known impending shortages is modest. By definition, the drug shortage crisis experienced around Europe is a failure of pharmaceutical companies to fulfil their public service obligations.

Although early notification seems not to be in interests of individual companies facing a shortage, a working early notification system could benefit the pharmaceutical industry as a whole by enabling companies to better predict changes in demand. Promotion of more responsible practices could become a concern for industry organisations also.

The temporary introduction of progressive fees in Finland demonstrated that even a modest but systematic and consistent economic incentive could improve companies’ commitment to timely notification(s). Although results of this natural experiment were still far from satisfactory, it provides a valuable lesson for future regulatory efforts.

The EU Commission published a proposal for a new EU medicines regulation in April 2023 [27]. The new regulation would lengthen the lead time for the market holder’s advance notification obligation to 6 months and add 12 months advance notification obligation for withdrawals. It would also add the obligation for companies to have a shortage prevention plan for every drug product in the market. Our results underline the need for adequate oversight and enforcement to realise these obligations.

### State of shortage registers

Compared to our 2020 study [23], we can see that public shortage notification systems in EU/EEA countries are developing and harmonising. However, member countries must continue to improve their drug shortage registers if these registers are to serve their intended functions.

There were dramatic differences in the number of shortage notifications among the country registers studied. This is consistent with prior research findings of widespread nonreporting of shortages to national authorities [10, 24–26]. On the other hand, there are differences in registration practices which may also affect the number of shortage notifications. In some countries, for example, shortages of different package sizes of the same drug product are registered as separate shortages, while in some countries they are reported as one shortage.

Looking at shortage registers, the difference between discontinuation and a temporary stoppage of supply could be more precise. In some cases, the stated rationale in the shortage notifications was “commercial” or “low sales”, which are typical reasons for product discontinuation. On the other hand, some market withdrawal notifications were attributed to production problems, a typical reason for a temporary shortage. Additionally, some cases gave both commercial and other reasons.

### Earlier research and study limitations

To the best of our knowledge and search efforts, there is barely any earlier research on actual notification times of drug shortages.

Vogler & Fischer (2020) charted drug shortage policies in different European countries [17]. According to that study, sanctions for failure to notify about upcoming shortages were possible in Germany and Slovenia, though in Sweden and Norway sanctions reportedly did not exist, and information about sanctions was unknown for Belgium, Croatia and Slovakia. These differences did not seem to be associated with the notification times we found in the registers, perhaps due to inconsistent enforcement.

Most of the 25 national medicine authorities did not publish notification dates of the shortages. However, the similarity of results across the eight countries studied suggests that results could be generalised at least in the EU area*.*

In Norway and Croatia, the inclusion of possible product withdrawal notifications probably improved notification time results for these countries. The main limitation of this study is censoring, that is, the absence of drug shortages data for which there is no public notification. There is currently no reliable estimate of how many shortages are unreported.

## Conclusion

The EU regulatory requirement enacted in 2019 for early notification of drug shortages has been unsuccessful in eight member countries having openly available statistics for follow-up. The national policy experiment in Finland with a progressive notification fee seemed to increase its compliance with the early notification.

A functioning advance notification system would require reliable regulatory oversight and enforcement. Implementation of corporate responsibility and transparency in drug shortages should be a common goal for all national authorities, patient and professional organisations and manufacturers themselves.

It remains to be seen whether there will be sufficient public demands on the European and national levels for this to happen.

## Data Availability

The original data are public and obtainable from national medicines authorities: Belgian Federal Agency for Medicines and Health Products (FAMHP), Agency for medicinal products and medical devices of Croatia (HALMED), Finnish Medicines Agency (Fimea), German Federal Institute for Drugs and Medical devices (BfArM), Norwegian Medicines Agency, and Slovakian State Institute for Drug Control (SUKL), The Agency for Medicinal Products and Medical Devices of the Republic of Slovenia (JAZMP) and Swedish Medical Products Agency (Läkemedelsverket). The datasets analysed during the current study are also available upon reasonable request to the corresponding author.

## Abbreviations

EU: European Union
EU/EEA: European Union/European Economic Area. European Economic Area consists of 28 European Union countries and Iceland, Lichtenstein, and Norway
